# Personalised drug repositioning for Clear Cell Renal Cell Carcinoma using gene expression

**DOI:** 10.1038/s41598-018-23195-8

**Published:** 2018-03-27

**Authors:** Karel K. M. Koudijs, Anton G. T. Terwisscha van Scheltinga, Stefan Böhringer, Kirsten J. M. Schimmel, Henk-Jan Guchelaar

**Affiliations:** 1 Department of Clinical Pharmacy & Toxicology, Leiden University Medical Centre, Leiden, The Netherlands; 2 Department of Medical Statistics, Leiden University Medical Centre, Leiden, The Netherlands

## Abstract

Reversal of cancer gene expression is predictive of therapeutic potential and can be used to find new indications for existing drugs (drug repositioning). Gene expression reversal potential is currently calculated, in almost all studies, by pre-aggregating all tumour samples into a single group signature or a limited number of molecular subtype signatures. Here, we investigate whether drug repositioning based on individual tumour sample gene expression signatures outperforms the use of tumour group and subtype signatures. The tumour signatures were created using 534 tumour samples and 72 matched normal samples from 530 clear cell renal cell carcinoma (ccRCC) patients. More than 20,000 drug signatures were extracted from the CMAP and LINCS databases. We show that negative enrichment of individual tumour samples correlated (Spearman’s rho = 0.15) much better with the amount of differentially expressed genes in drug signatures than with the tumour group signature (Rho = 0.08) and the 4 tumour subtype signatures (Rho 0.036-0.11). Targeted drugs used against ccRCC, such as sirolimus and temsirolimus, which could not be identified with the pre-aggregated tumour signatures could be recovered using individual sample analysis. Thus, drug repositioning can be personalized by taking into account the gene expression profile of the individual’s tumour sample.

## Introduction

While targeted therapies such as tyrosine kinase inhibitors (sunitinib, sorafinib, pazopanib, axitinib, tivozanib) and mammalian target of rapamycin (mTOR) inhibitors (everolimus, sirolimus, temsirolimus) have greatly improved the prognosis of metastatic Clear Cell Renal Carcinoma (ccRCC) patients, the average duration of disease control ranges between 8–9 months in the first-line setting and 5–6 months in the second-line setting^1^. This is caused by intrinsic and/or acquired drug resistance^2^. Both are likely enhanced by the existence of inter- and intra-tumour molecular heterogeneity: a recent study demonstrated that different biopsies from the same ccRCC tumour grown in patient-derived xenograft (PDX) mouse models can show different drug sensitivity patterns, and each was associated with markedly different gene expression profiles^3^.

To prevent and overcome drug resistance, model systems and clinical experience have shown that combining drugs which target different pathways are superior to single-agent approaches^4^. However, combining oncological drugs also tends to prohibitively increase the toxicity, as evidenced by the use of sunitinib and everolimus simultaneously^5^. Therefore, to design better tolerated and effective combination regimens it might be productive to widen the search to include non-oncological drugs, as they often have a better safety profile. This is not as far-fetched as it may initially seem: aspirin, metformin, itraconazole and many other regular drugs are currently being tested in clinical trials for efficacy in adult malignancies, usually in combination with regular treatments^6^.

The application of already registered drugs and compounds for new indications is called drug repositioning and it has obvious appeal: knowing the safety, toxicity, pharmacokinetic, pharmacodynamic and metabolic properties of a compound significantly reduces the risk and time required to register an indication as compared to a new chemical entity^7^. This process has been accelerated by the surge of freely available “omics” data which has inspired many researchers to develop computational drug repositioning methods^8^. One popular method, gene expression signature reversal, is based on the observation that when the difference in gene expression of cells after perturbation by a compound (the drug signature) is negatively correlated to the difference in gene expression between diseased and normal cells (the disease signature), the drug often turns out to be therapeutically indicated for that disease^9^.

Because of the considerable heterogeneity between and even within ccRCC tumours, it therefore makes sense to reposition drugs based on individual gene expression profiles, as each tumour sample may have a different set of perturbed pathways^10^. If all samples are analyzed collectively, pathways could either be masked or be less prominently expressed in proportion to the incidence of the perturbation. One way to solve this problem is to divide the tumour gene expression signature into subtypes with a statistical technique such as hierarchical clustering, which groups samples with similar expression profiles. If no real biological variability remains within the identified subtypes (i.e. the same pathways are similarly perturbed in all samples belonging to that subtype), the probability of finding a valid result increases due to increased power as bigger groups are compared. However, if subtypes do contain significant biological variability it could interfere with the correct identification of potentially therapeutic drugs. Analyzing individual tumour samples does not suffer from this potentially incorrect binning problem, and could therefore result in more potentially therapeutic drug hits and simultaneously provide information on the proportion of tumour samples which are negatively correlated to each drug signature, at the cost of reduced power when samples are homogeneous. Although development of a method to target drugs based on the gene expression of single tumour samples may not be successful for all tumours due to the existence of inter and intra-tumour heterogeneity, however, such an approach would best approximate the situation of intra-tumour heterogeneity and could be extended to multiple tumour samples in the future.

The goal of this research is to benchmark the results of the gene expression reversal analysis of the tumour subtype and the individual tumour sample signatures against the results of the average ccRCC tumour signature, as this could provide support for the development of an individualized drug repositioning approach based on gene expression.

## Results

### Clear Cell Renal Cell Carcinoma expression profiles

In total, 610 expression profiles from 606 different tissue samples (of which 72 matched solid tissue normal) were included in the analysis (Table 1). The ‘new primary solid tumour tissue sample’ (i.e. the metastasis of an earlier ccRCC tumour) was excluded from further analysis, as it could skew the results from the far more common 534 original primary solid tumour samples. These tissue samples were taken from 530 different patients, 344 male and 186 female. Of 4 patients (3 in stage I & 1 in stage II), 2 tumour samples were taken and one of these was analysed twice. The age of the patients ranged from a minimum of 26.6 years to a maximum of 90 years with a median age of 61 years.

**Table 1:** Tissue samples.

Tissue	Subgroup	Samples
Primary solid tumour	Stage I	268
	Stage II	58
	Stage III	123
	Stage IV	82
	Unknown	3
	Total	534
Matched solid tissue normal	Total	72
New primary solid tumour	Total	1

**Figure 1:**
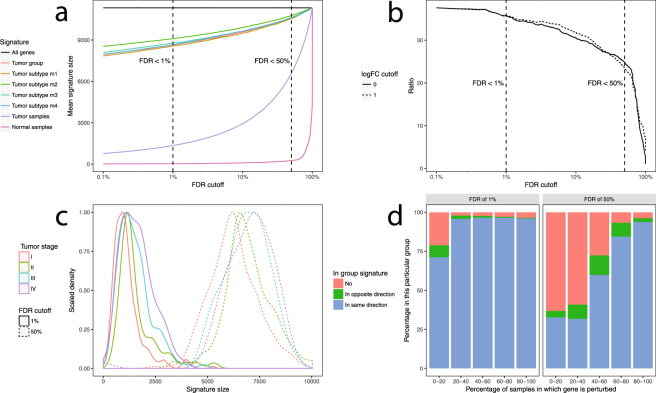
(**a**) Signature sizes of tumour group, tumour subtypes, tumour samples and normal samples plotted against FDR cutoff. (**b**) Ratio of average tumour sample signature size divided by average normal sample signature size plotted against FDR cutoff. (**c**) Distribution of tumour sample signature sizes by tumour stage at an FDR cutoff of 1% and 50%. (**d**) Inclusion and directionality of genes plotted against perturbation frequency at an FDR cutoff of 1% and 50%.

**Figure 2:**
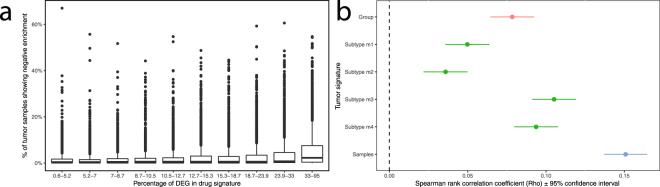
(**a**) Amount of samples showing statistically significant enrichment versus % of DEG in LINCS drug signature across the 10 deciles of ±1940 compounds. (**b**) Correlation between negative enrichment frequency of signature types versus % DEG in drug signature.

**Figure 3:**
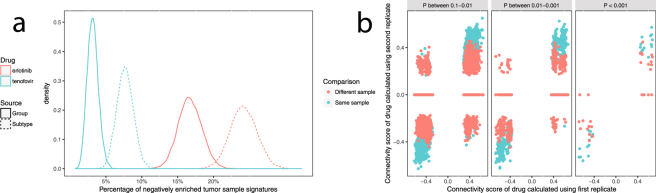
(**a**) Density plot of negative enrichment frequency with erlotinib and tenofovir of 10,000 simulated batches of 530 tumour samples assuming they were sourced from the tumour group signature (solid lines) or subtype signatures (dashed lines). (**b**) Connectivity scores of analytical replicates on the same 4 tissue samples (green) and from 4 different tissue samples from the same 4 patients (red) plotted against each other, for three P cutoff intervals.

After filtering out low expressed genes, the genes remaining in the analysis were reduced from 23,247 to 11,333 (−51%). Varying the CPM cutoff or minimum sample requirement around these values does not significantly affect the number of genes remaining in the analysis (Figs 1,2 Supplement). Furthermore, no substantial batch effects were observed as assessed by Principal Components Analysis (Fig. 3 Supplement), conform the original analysis of the data by TCGA^11^.

### Tumour signatures

Fig. 1a illustrates that although the signature sizes of the tumour samples are smaller than those of the tumour group and subtypes, the signatures sizes of the tumour samples are many times bigger than those of the normal samples at any false discovery rate (FDR) < 100%. This contrast is even clearer to see in Fig. 1b: up to an FDR of 50% the amount of differentially expressed genes compared to the control condition (i.e. each individual normal sample versus all the other samples), remains at minimum 20 times higher. Fig. 1c further emphasizes that there’s information in the individual signatures, as the amount of differentially expressed genes (DEG) increases monotonically with tumour stage. Lastly Fig. 1d shows that at the more liberal FDR cutoff of 50%, sizeable fractions of genes frequently differentially expressed in the individual samples are in the opposite direction of the tumour group signature containing genes with a FDR < 1%.

### Drug signatures

Of the 1,309 drugs tested in CMAP and the 19,812 drugs tested in LINCS, 21 (1.6%) and 388 (2.0%) could not be processed further because the linear model could not be fitted due to the lack of control samples. The genes measured by the CMAP and LINCS arrays shared 6,058 and 502 genes in common with the 11,333 genes included in the tumour gene expression analysis, respectively (Fig. 4 supplement). However, LINCS drug signatures contain on average 6 times more genes with a FDR below 50% based on the set of 879 genes shared between CMAP and LINCS when tested on the shared set of 979 drugs (Fig. 5 Supplement).

### Connectivity mapping

The tumour sample signatures show a much higher rate of negative enrichment when connectivity mapping to the LINCS drug signatures than with the CMAP drug signatures (P < 10^−16^, Wilcoxon rank sum test). Furthermore, the amount of DEG in a drug signature (a marker of signature quality) shows a much stronger correlation with tumour sample negative enrichment rate (Fig. 2a, Spearman’s Rho = 0.15, P < 10^−16^) than with the amount of negatively enriched drugs calculated with the group and subtype signatures (Spearman’s Rho = 0.08 for group signature, between 0.036–0.11 for subtype signatures, Fig. 2b). The mTOR inhibitors sirolimus (P = 0.03) and temsirolimus (P = 0.004) show negative enrichment with the individual tumour sample signatures, but not with the tumour group or subtype signatures. In contrast, the tyrosine kinase inhibitors approved for use against ccRCC (axitinib, pazopanib, sorafenib, sunitinib) do not show statistically negative enrichment with any signature type.

The top 8 results of connectivity mapping the 19,424 LINCS drug signatures to the 530 first tumour samples taken from each patient after filtering out drugs not clinically available are presented in Table 2. Diverse classes of drugs are represented, most of which anti-tumour activity against ccRCC was not expected a priori.

**Table 2:** Top 8 LINCS drugs in clinical use which show the most frequent negative enrichment of tumour samples and which have >33% differentially expressed genes, all with a P value < 0.01 and a FDR <10%.

Drug	% of samples	Mechanism of action	Current indications
erlotinib	45	A tyrosine kinase inhibitor for the EGFR receptor.	Primarily used in non-small cell lung cancer and pancreas carcinoma.
elvitegravir	41	An integrase inhibitor.	HIV infection.
tenofovir	39	Nucleotide reverse transcriptase inhibitor.	Chronic hepatitis B and prevention/treatment HIV/AIDS.
trimidox (trimethoprim + sulfadoxine)	36	Inhibition of dihydrofolate reductase, reduces folic acid	Bacterial infections.
nicotinamide	30	Part of the vitamin B3 complex. Has anti-inflammatory properties.	Niacin deficiency, Acne.
quinine	29	Inhibition of hemozoin biocrystallization of parasites	Malaria and babesiosis.
genistein	26	Supposedly many, e.g. inhibition of EGFR and DNA topoisomerase.	None registered, used as a dietary supplement.
temsirolimus	24	Inhibition of mammalian Target Of Rapamycin.	Clear cell renal cell carcinoma.

If the tumour samples are simulated based on the tumour group signature, the expected amount of negatively enriched tumour samples in 95% of cases would be at least 2.3 up to 3.2 times lower for erlotinib, a drug which is negatively correlated to the group signature at P < 0.01, and 8.7 up to 21 times lower for tenofovir, which is only slightly negatively correlated to the group signature with P = 0.15 (Fig. 3a). If the samples are simulated from a representative distribution of tumour subtypes, then the difference becomes a little smaller: 95% of simulated batches return 1.6 up to 2.1 less negatively enriched tumour samples with erlotinib, and 4.1 to 6.7 times less for tenofovir. This same pattern, i.e. the samples simulated from the subtype signatures moving closer to the actual individual negative sample enrichment rate, was observed for the other 6 drugs (Fig. 6 Supplement). Lastly, Fig. 3b illustrates the directional consistency of the connectivity scores when the same 4 samples are analyzed again contrasted with the result of connectivity mapping the tumour sample signatures of 4 different samples from the same patients. At P value intervals between 0.1–0.01, between 0.01–0.001 and below 0.001, there’s a respectively 76%, 87% and 96% probability the connectivity score has the same sign upon re-analysis of the same sample. However, when the same P value intervals but different samples from the same patient are used, these probabilities drop to respectively 50%, 61% and 78%.

## Discussion

In this study we showed that an individual analysis of tumour samples result in more potentially therapeutic drug hits which are negatively correlated to each drug signature. This supports the development of an individualized drug repositioning approach based on gene expression.

Despite the fact that none of the drugs were tested on any ccRCC cell lines, connectivity mapping of the tumour sample signatures (but not the group or subtype signatures) does reveal significant negative enrichment for 2 out of the 3 mTOR-inhibitors used against ccRCC (sirolimus and temsirolimus). However, the other mTOR inhibitor (everolimus) and the 4 tyrosine kinase inhibitors used against ccRCC present in the LINCS database (axitinib, pazopanib, sorafenib, sunitinib) did not reveal significant negative enrichment. This could be because these drugs were tested in fewer cell lines (N = 13–20) as compared to sirolimus and temsirolimus (N = 51–57). Furthermore, these tyrosine kinase inhibitors are believed to work because they primarily inhibit the Vascular Endothelial Growth Factor (VEGF) receptor present on non-cancerous endothelial cells^1^, and therefore reversal of gene expression on the ccRCC cells is not expected to occur.

Our study also shows that the approach of drug repositioning by gene expression reversal reveals interesting potential drugs for treatment of individuals with ccRCC. Indeed, most of the top 8 of drugs with the highest negative enrichment of tumour sample signatures are already supported by existing evidence. Erlotinib and genistein both inhibit the Endothelial Growth Factor Receptor (EGFR), and the EGFR gene expression profile showed overexpression in 38.2% of tumour samples from an independent cohort of 63 ccRCC patients^12^. The nucleotide reverse transcriptase inhibitor tenofovir is associated with nephrotoxicity due to accumulation in the proximal tubules^13^, which ccRCC is thought to originate from. The similarity in gene expression between ccRCC and proximal tubules cells has been noted before^14^, and therefore it seems plausible they share the same toxicity as well. Quinine has shown some efficacy as an add-on in breast cancer patients^15^ and nicotinamide has been shown to substantially reduce the recurrence risk of skin cancer in a RCT^16^. Lastly, temsirolimus is already in use against ccRCC.

Some potential statistical issues/refinements of the described pipeline remain: the current method of determining the drug signature equally weighs experimental instances equally with different drug concentrations and drug exposure durations, whereas it has been demonstrated that higher drug concentrations and exposure durations induce a stronger effect on the differential gene expression profiles^17^. More sophisticated batch effect correction methods than including a factor in the linear model exist, *e.g*. the use of control genes, could further amplify the signal from the noise^18^. Different connectivity scoring methods also exist, which could further improve the sensitivity and/or specificity of the pipeline^19^. Lastly, the selection of tumour sample genes was done by the commonly accepted but arbitrary criterion of a FDR below 1%; ideally this cut-off would be determined from the data, or genes more likely to be actually differentially expressed could be given a higher weight. It will be more difficult to quantify the false negative rate which can be increased by biological factors, e.g. if drugs are tested in cell lines which do not express the drug target(s). However, as the amount of hits that can be validated is likely small, perhaps the focus should mainly be on decreasing the false positive rate.

Despite all these potential issues/refinements however, the simulations already make it very clear that it would have been extremely unlikely to have found the same results if all tumour samples came from a single uniform tumour expression profile or a representative combination of the 4 previously identified subtype profiles. Indeed, the power of this approach lies in not having to assume the number of subtypes, whether there are none, 4 or more. Repeated RNA-seq analysis of the same tissue sample, and to a lesser degree a new tissue sample from the same patient, already shows remarkable consistency in connectivity scores calculated with the current method and increases as the P-value of the connectivity score decreases.

To our knowledge, this is the first paper to convincingly demonstrate that using individual tumour sample signatures as the basis for analysis outperforms analyses based on tumour group or subtype signatures. Zerbini *et al*. did publish a similar analysis^10^, but as this was the only type of analysis they did, it did not demonstrate the superiority to the connectivity mapping of the group or subtype tumour signatures. Furthermore, our analysis contains more than 25 times more, and arguably better characterized, tumour samples and more than 120 times more drug compounds profiled in far more cell lines, resulting in a more comprehensive analysis.

Whether a pathway is causally involved in the survival of the tumour is impossible to determine from the gene expression data alone. Testing the hits on existing ccRCC cell lines is a possibility, but many aspects of ccRCC cannot be replicated using cell lines^20^. Furthermore, the transcriptomic heterogeneity is unlikely to be well represented in existing cell lines. It could be an option to first grow some of the patient’s own tumour tissue in an and test the drugs using an *ex vivo* functional assay. When the candidate drugs are marketed drugs which have been tested and approved for clinical use within the dose range an effect can be expected, it might therefore be ethically defensible to directly prescribe the drug off-label in an N = 1 trial. Possibly both scenarios could even be run in parallel, with the *ex vivo* results providing some external validation and validated alternatives in the event the first prescription failed to provide any benefit. If a database of the results of these N = 1 trials can be created and maintained, it could eventually generate enough evidence to conclude which combinations of gene expression profiles and drugs show a consistent therapeutic benefit.

## Materials and Methods

All data processing and analyses were performed using R version 3.4.0 using the R/Bioconductor packages mentioned below. FDR was always calculated using the Benjamini-Hochberg method.

### Clear Cell Renal Cell Carcinoma expression profiles

The mRNA-seq v2 read counts produced by The Cancer Genome Atlas (TCGA) Kidney Renal Clear Cell Carcinoma project were downloaded from the Genomic Data Commons using the TCGAbiolinks package (version 2.5.7)^21^. It is automatically annotated with metadata, such as patient information and the molecular subtype as determined by the original TCGA analysis using hierarchical cluster analysis^11^. The genes were normalized within samples by gene length and between samples to correct for sequencing depth using the EDAseq package (version 2.10.0)^22^. Only genes which were expressed above 0.5 Counts Per Million (CPM) in at least a sixth of all samples were retained, i.e. selection was irrespective of tissue type. This cutoff was taken from a Bioconductor example workflow, and reduces the high noise inherent in measuring lowly expressed genes^23^.

### Tumour signatures

The read counts were modelled using the negative binomial generalized log-linear model available from the edgeR package (version 3.18.1)^22^, and statistical significance was assessed using a likelihood ratio test. Three different types of tumour signature were generated:

1. Tumour group signature: All tumour samples versus all normal samples. This is the conventional comparison, and ideal under the assumption of homogenous tumours.
2. Tumour subtype signatures: All tumour samples belonging to each one of the 4 molecular tumour subtypes versus all normal samples.
3. Tumour sample signatures: Each tumour sample versus all normal samples. This provides a unique signature for each tumour sample.

To estimate the true false positive rate of genes included in the tumour sample signatures, normal sample signatures were generated by comparing each normal sample versus all the other normal samples.

### Drug signatures

A preprocessed version of the CMAP database was downloaded using the PharmacoGx package (version 1.6.1) which was corrected for systematic differences caused by the different microarray platforms using the ComBat function in the sva package (version 3.10.0)^24^.

The LINCS database was downloaded directly from the Gene Expression Omnibus (GSE92742) in the Level 3 format. The Entrez gene identifiers were converted to Ensembl gene identifiers using the bioMart package (version 2.32.1), to make them compatible with the tumour expression profiles and the preprocessed CMAP database^25^.

The drug signatures were calculated with a linear model using the limma package (version 3.32.5) with drug concentration as a linear parameter (i.e. 0 for controls and >0 for drugged cells) and cell type, perturbation duration (if >2 perturbation durations), batch as categorical variables^26^

### Drug-tumour signature connectivity mapping

Gene Set Enrichment Analysis (GSEA), using the log2 fold difference of the tumour genes which are below a 1% FDR in combination with using the landmark genes and tumour cells resulted in the least amount of false positive results out of all tested methods and configurations, when CMAP drug signatures were used to retrieve the corresponding LINCS drug signatures (Figures 7–10 Supplement). Therefore, the same configuration was used for the drug-tumour signature connectivity mapping.

Negative enrichment is defined as a negative connectivity score with an associated P-value below 0.05. P-values were calculated using permutations; N = 1000 for each sample and N = 1000 * the amount of tumour samples for the group and subtype signatures. P-values of cumulative tumour sample enrichment were determined by comparing the amount of negatively enriched samples to the distribution observed for drug signatures containing less differentially expressed genes (DEG) than the amount which would be expected by chance 95% of the time. FDR correction was applied separately for each decile of drug signature percentage DEG.

### Simulation study

For the top 8 most frequently negatively enriched drugs, a simulation study was performed to validate which drugs show individual differences in connectivity score different from the group and subtype signatures. 10,000 batches of individual tumour samples of the same size as the original batch and with the same distribution of sequencing depth to the original tumour samples were simulated from the tumour group signature to determine the amount of negatively enriched tumour sample signatures. Another 10,000 similarly constructed batches were sampled from the subtype signatures in the same proportion as found in the original data. Simulation was performed by extracting the mu and size parameters for each gene from the negative binomial generalized log-linear model. Tumour sample signatures were then calculated to determine the connectivity score and associated P-value with each drug.

### Data availability

The R code, drug signatures, tumour signatures and the resulting datasets generated during the current study are available in a public GitLab repository (https://gitlab.com/k.k.m.koudijs/personalised_DR_ccRCC).

## Electronic supplementary material

Supplementary figures
