# Supplementary material for: Personalised drug repositioning for Clear Cell Renal Cell Carcinoma using gene expression
Source: Sci Rep. 2018 Mar 27;8:5250. doi: 10.1038/s41598-018-23195-8 (PMC5869697; doi:10.1038/s41598-018-23195-8)
Supplement: Supplementary file 1 — Supplementary figures [file 41598_2018_23195_MOESM1_ESM.pdf]

Supplementary figures to 'Personalised drug repositioning for Clear Cell Renal Cell Carcinoma using gene expression'

Karel K.M. Koudijs[a], Anton G.T. Terwisscha van Scheltinga[a], Stefan Böhringer[b], Kirsten J.M. Schimmel[a], Henk-Jan Guchelaar[a*]
* = corresponding author

Affiliations:
a. Department of Clinical Pharmacy & Toxicology,
Leiden University Medical Center, The Netherlands
b. Department of Medical Statistics,
Leiden University Medical Center, The Netherlands

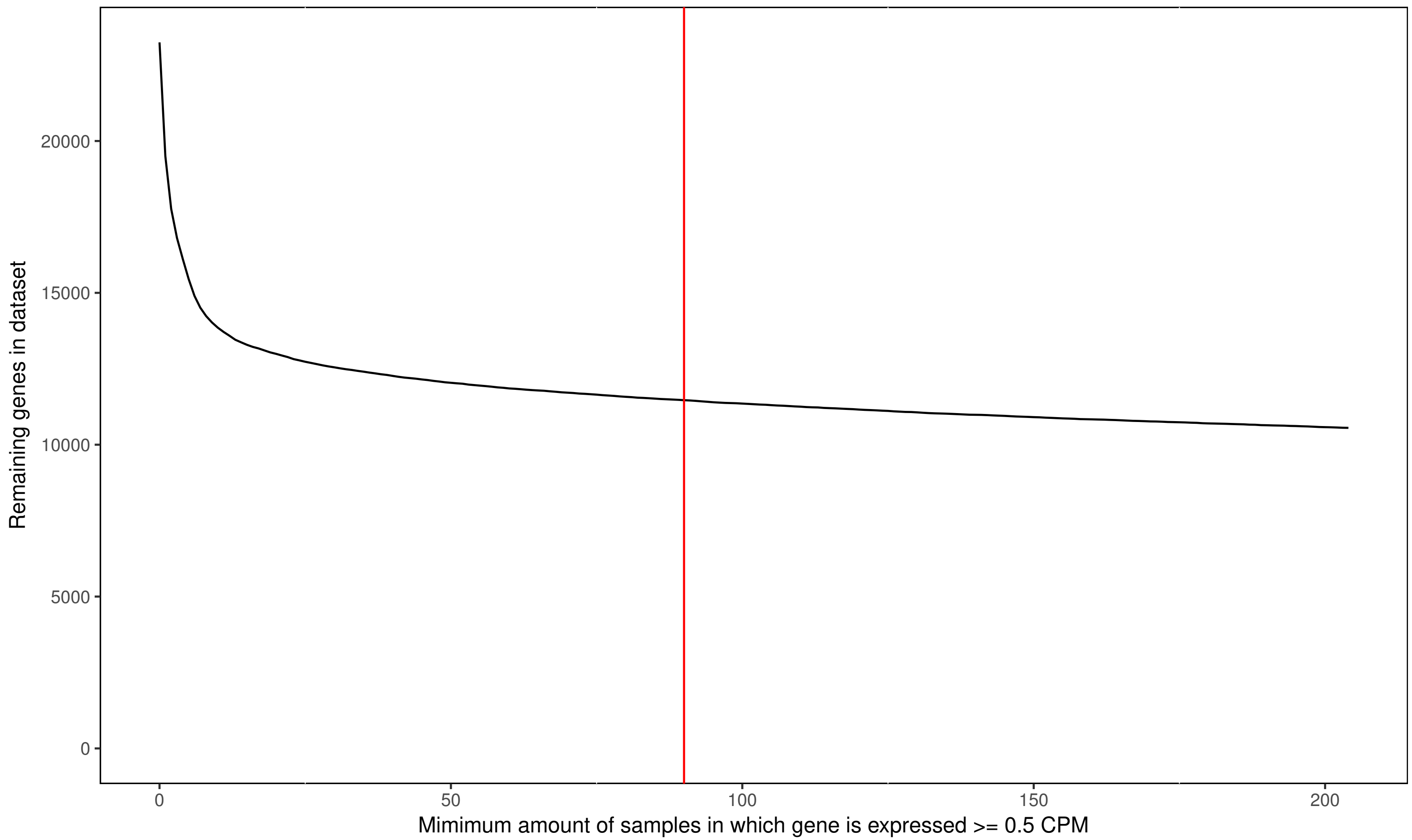

Supplementary Figure 1: Impact of varying minimum sample requirement at a static cutoff of 0.5 Counts Per Million (CPM). The red line is the chosen cutoff. The red line is the chosen cutoff resulting in 11,333 genes of the 23,247 remaining in the analysis.

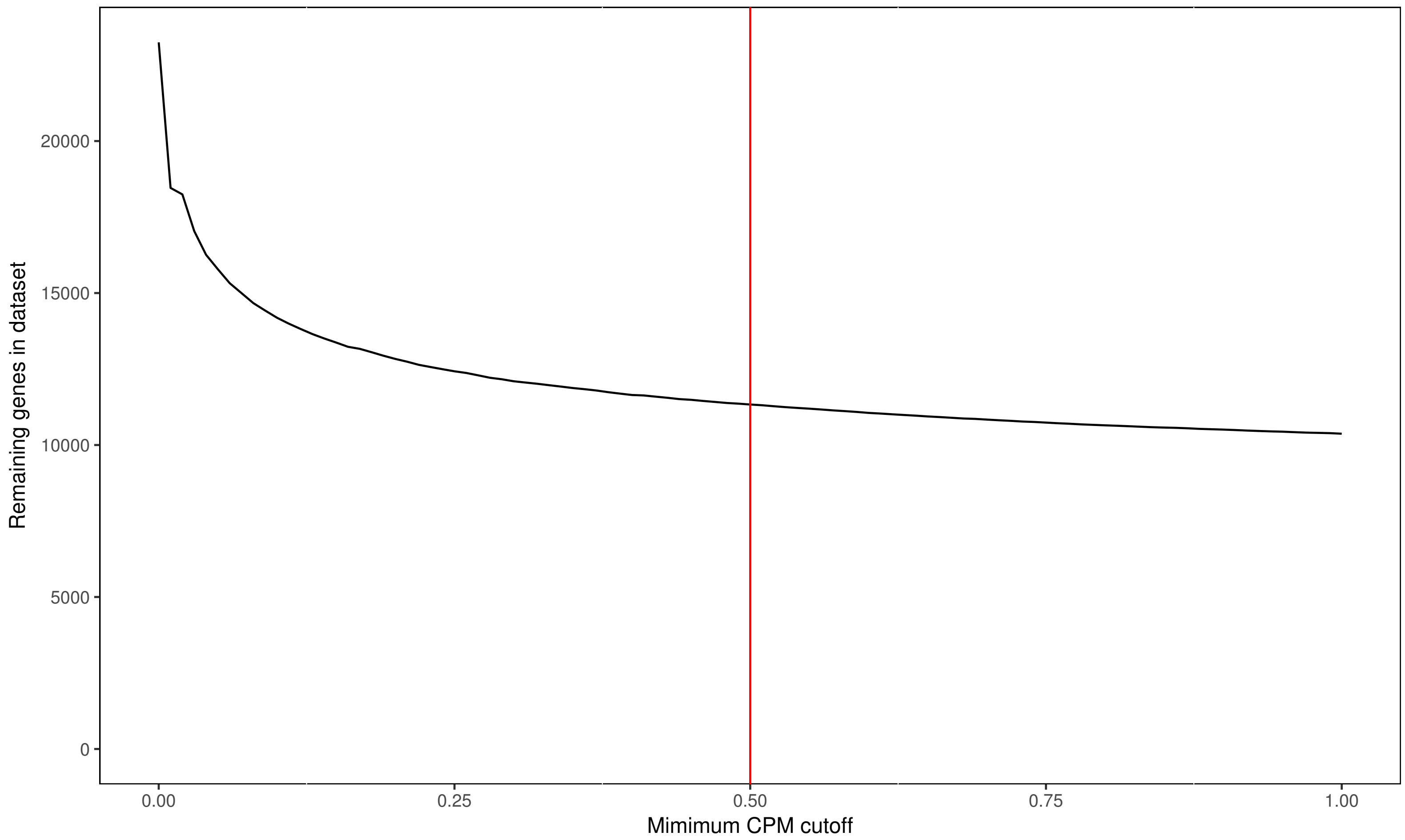

Supplementary Figure 2: Impact of varying minimum Counts Per Million (CPM) requirement at a static cutoff of expression in at least 90 samples. The red line is the chosen cutoff resulting in 11,333 genes of the 23,247 remaining in the analysis.

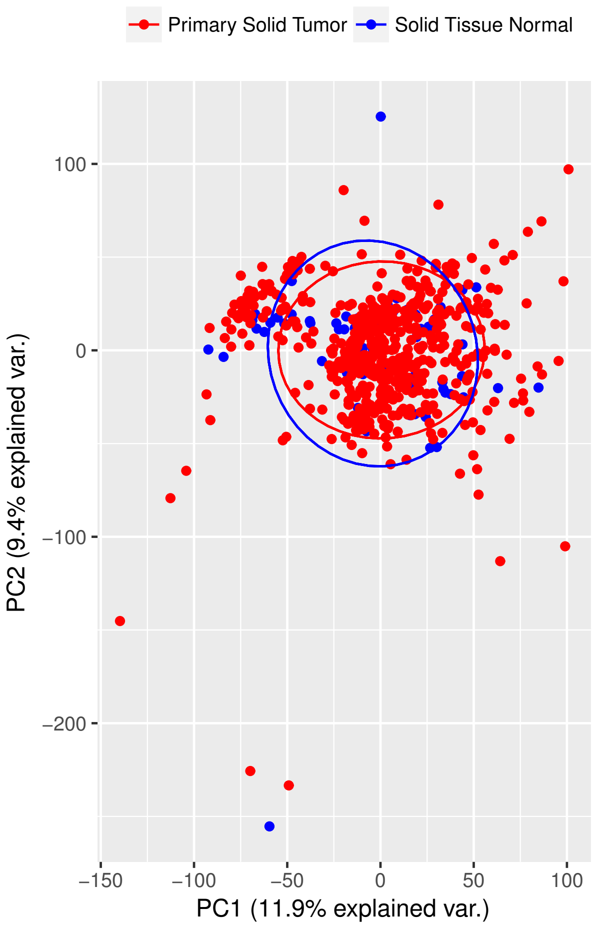

Supplementary Figure 3: Results of Principal Components Analysis (PCA) of the CPM transformed data.

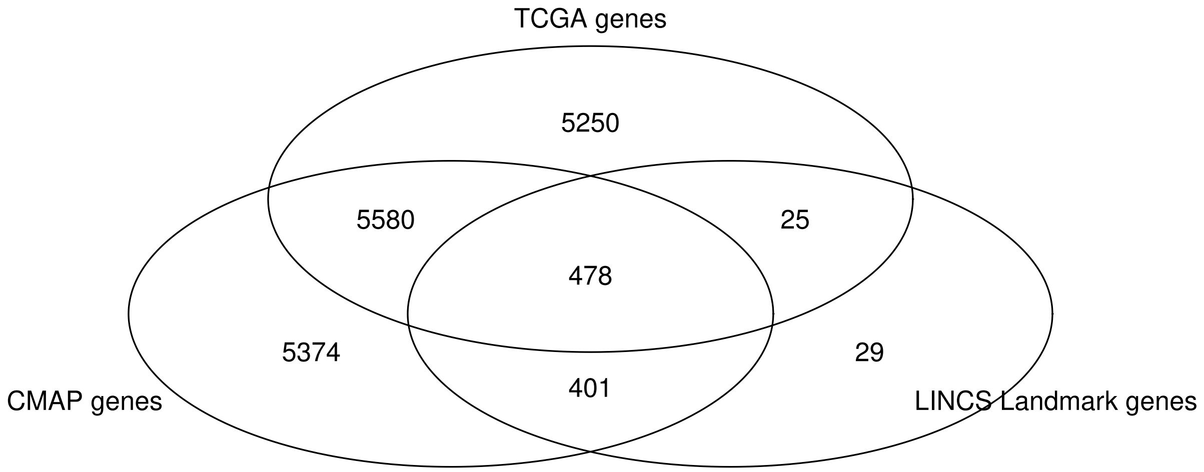

Supplementary Figure 4: Overlap of Ensembl gene identifiers between CMAP, LINCS and TCGA expression data

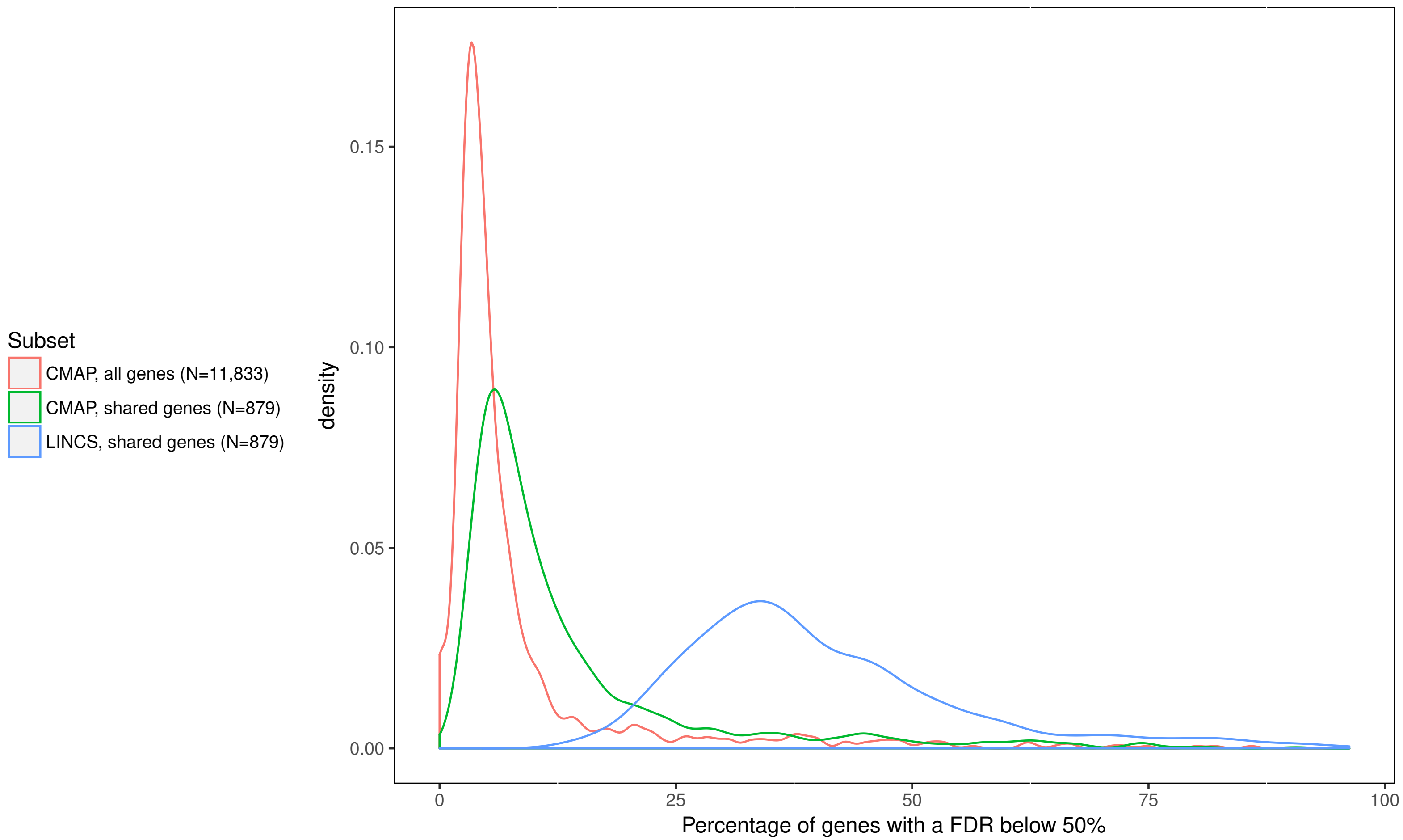

Supplementary Figure 5: Percentage of genes with a FDR below 50% for all CMAP genes, only the CMAP genes shared with LINCS and the LINCS genes which are also measured by CMAP.

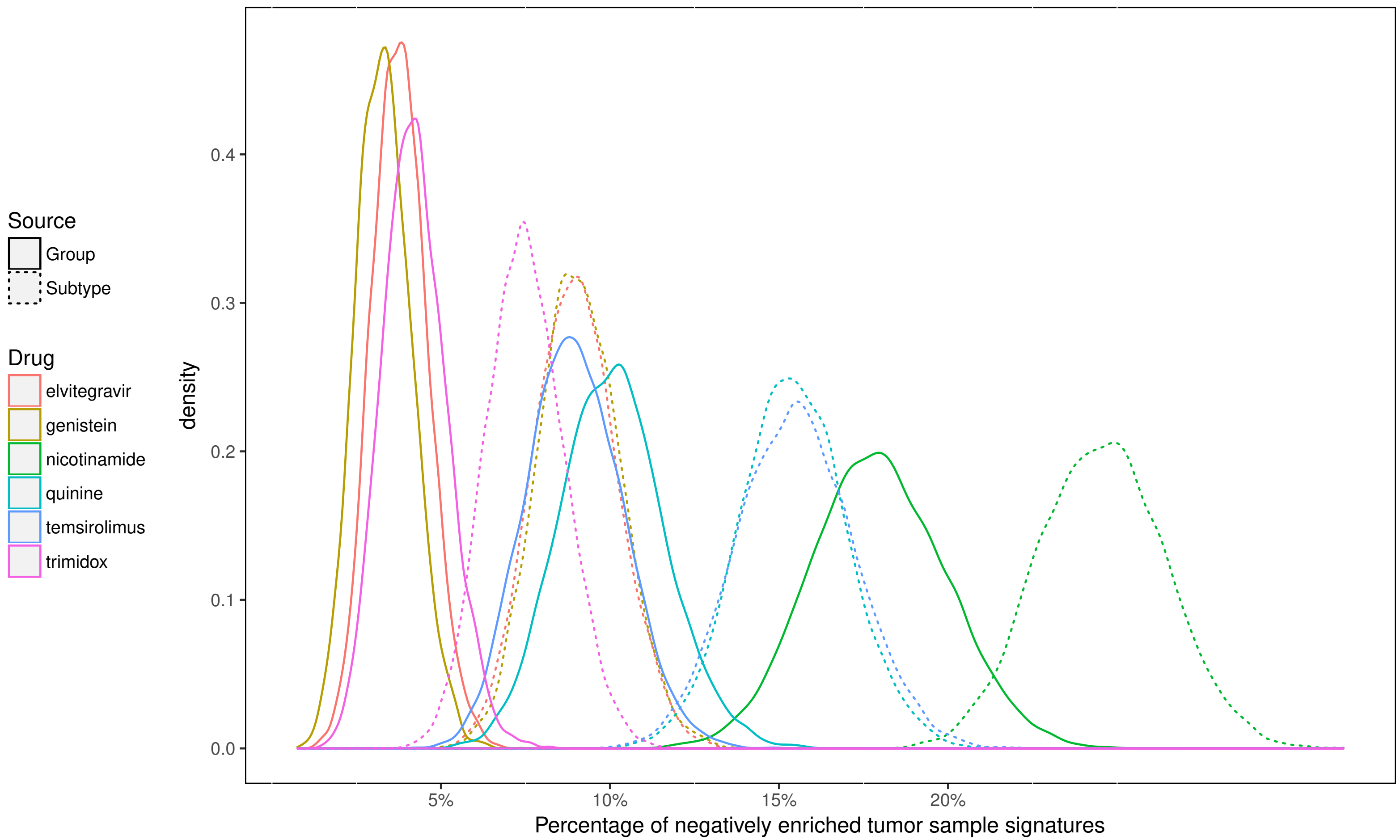

Supplementary Figure 6: Density plot of negative enrichment frequency with other top hits of 10,000 simulated batches of 530 tumor samples assuming they were sourced from the tumor group signature (solid lines) or subtype signatures (dashed lines).

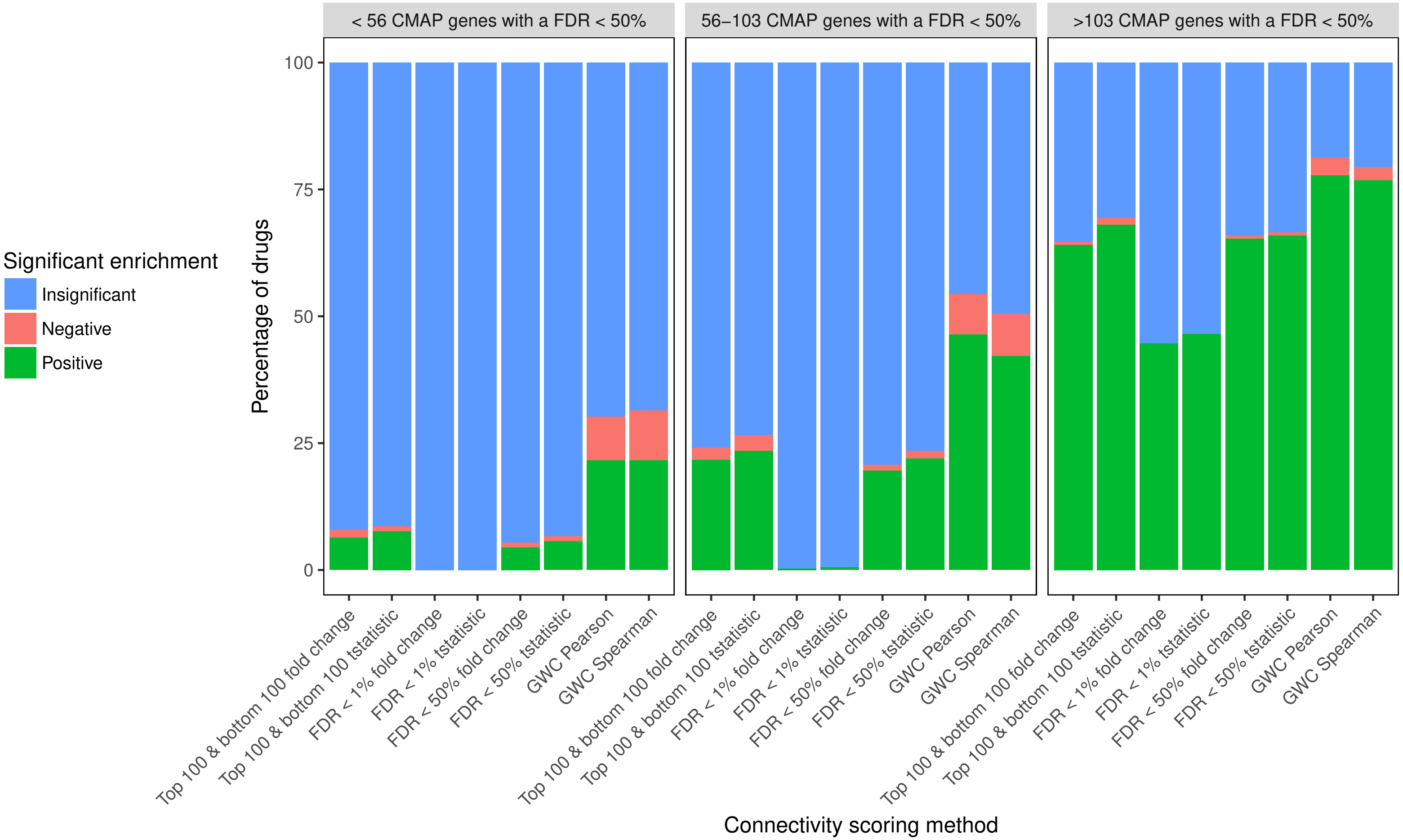

Supplementary Figure 7: Performance of benchmarked methods stratified across the 3 quantiles of genes with a FDR $< 50\%$

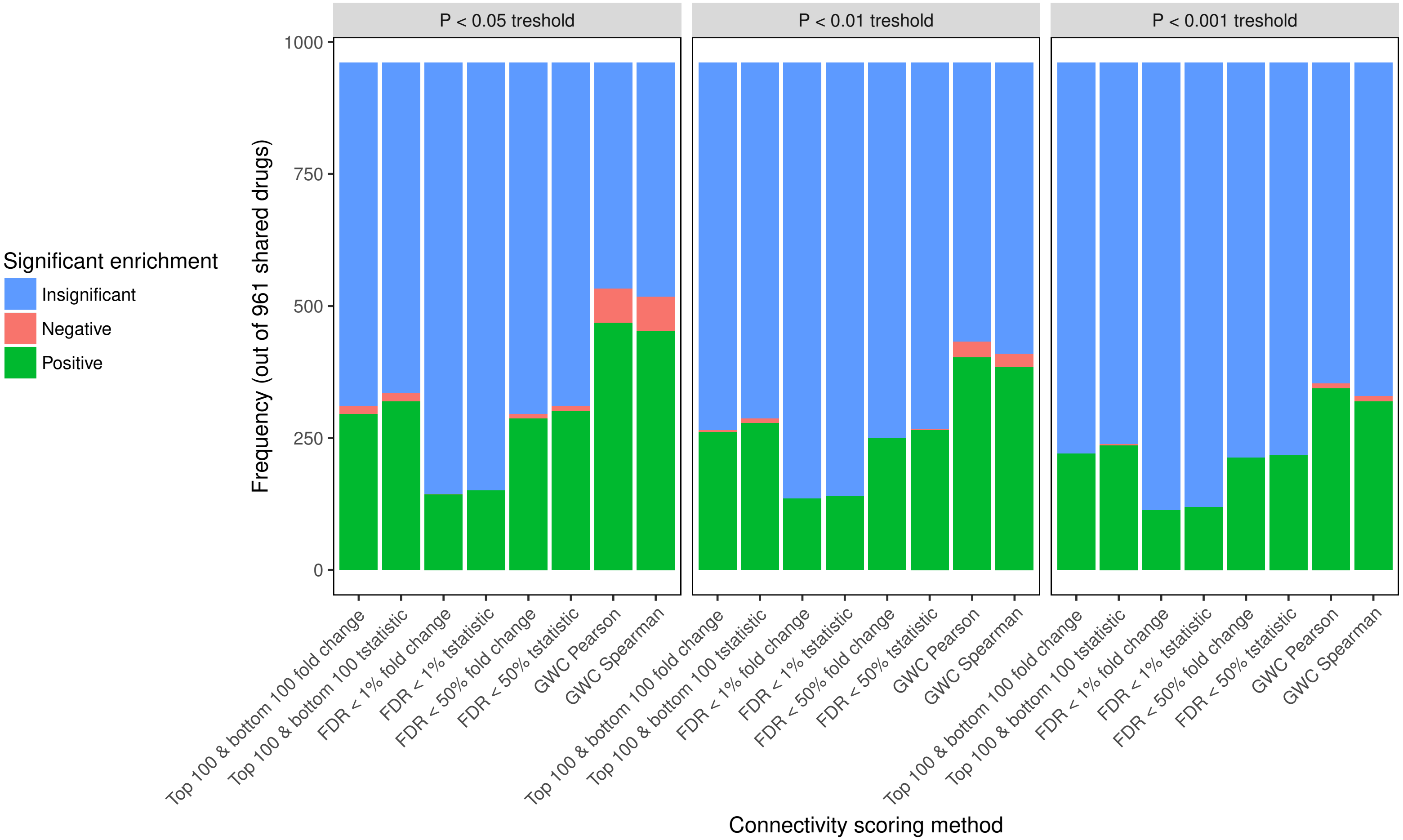

Supplementary Figure 8: Performance of benchmarked methods stratified across 3 cutoffs of critical P values

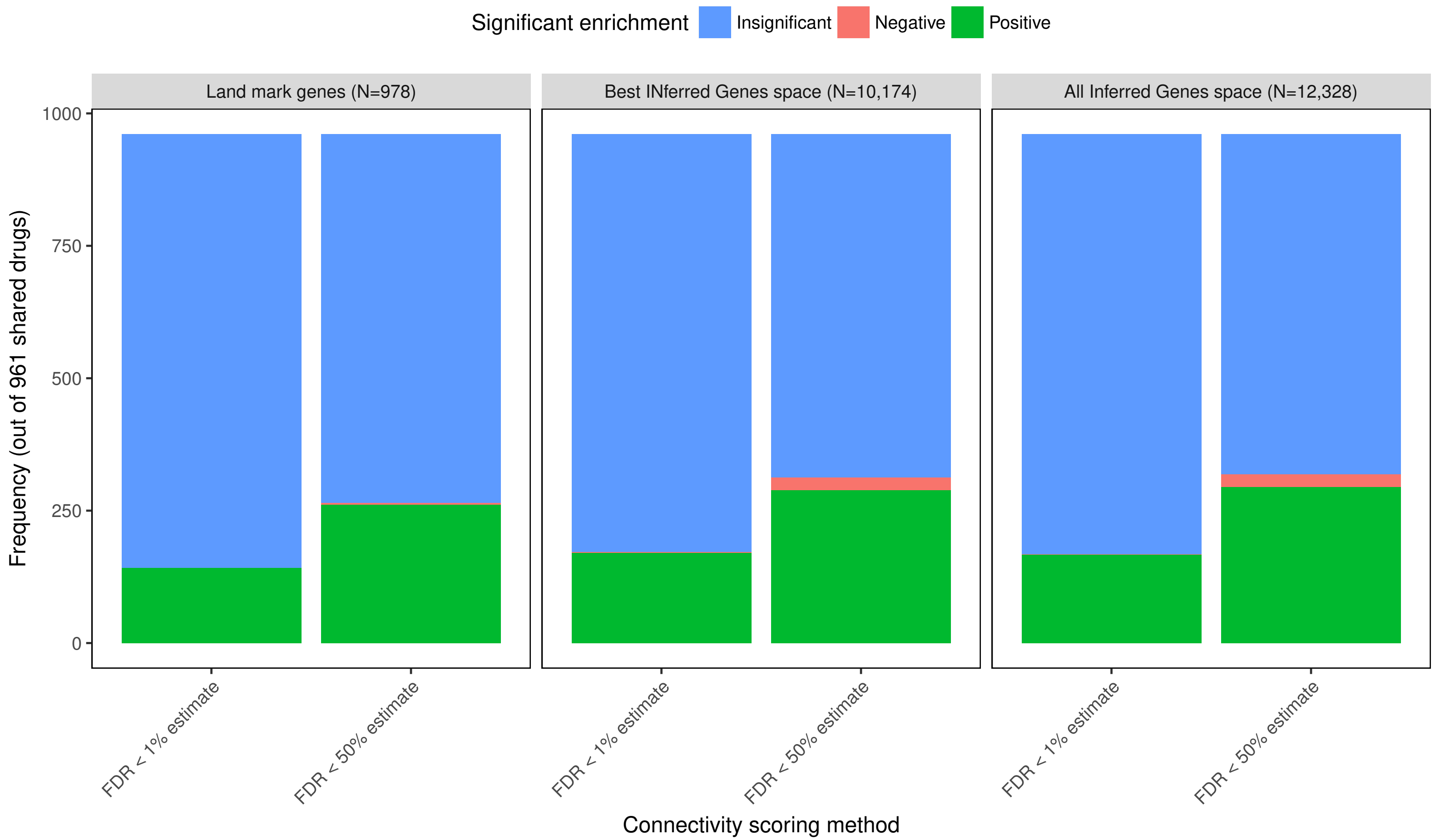

Supplementary Figure 9: Enrichment frequency of CMAP drugs against LINCS signatures of the same drug based on Landmark genes (LM), Best inferred genes (BING) and All inferred genes (AIG)

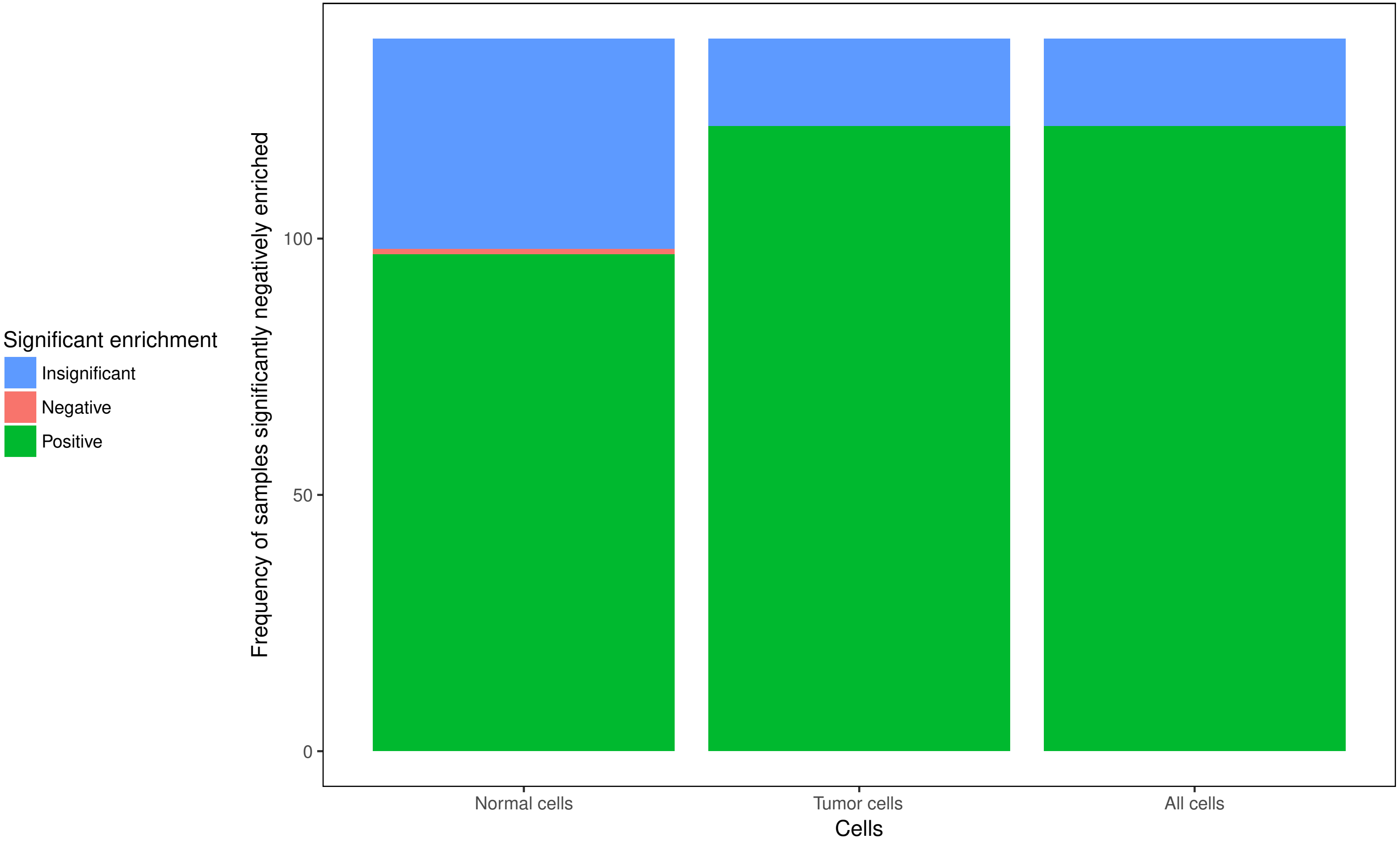

Supplementary Figure 10: Enrichment frequency of 139 CMAP drugs with at least 10 genes with a FDR < 1% and at least 3 LINCS samples in normal and tumor cells
